# Phanerozoic survivors: Actinopterygian evolution through the Permo‐Triassic and Triassic‐Jurassic mass extinction events

**DOI:** 10.1111/evo.13421

**Published:** 2018-02-02

**Authors:** Fiann M. Smithwick, Thomas L. Stubbs

**Affiliations:** ^1^ Department of Earth Sciences University of Bristol Bristol BS8 1TQ United Kingdom

**Keywords:** Actinopterygii, end‐Triassic extinction, mass extinctions, morphometrics, macroevolution, Permo‐Triassic

## Abstract

Actinopterygians (ray‐finned fishes) successfully passed through four of the big five mass extinction events of the Phanerozoic, but the effects of these crises on the group are poorly understood. Many researchers have assumed that the Permo‐Triassic mass extinction (PTME) and end‐Triassic extinction (ETE) had little impact on actinopterygians, despite devastating many other groups. Here, two morphometric techniques, geometric (body shape) and functional (jaw morphology), are used to assess the effects of these two extinction events on the group. The PTME elicits no significant shifts in functional disparity while body shape disparity increases. An expansion of body shape and functional disparity coincides with the neopterygian radiation and evolution of novel feeding adaptations in the Middle‐Late Triassic. Through the ETE, small decreases are seen in shape and functional disparity, but are unlikely to represent major changes brought about by the extinction event. In the Early Jurassic, further expansions into novel areas of ecospace indicative of durophagy occur, potentially linked to losses in the ETE. As no evidence is found for major perturbations in actinopterygian evolution through either extinction event, the group appears to have been immune to two major environmental crises that were disastrous to most other organisms.

The Actinopterygii (ray‐finned fishes) represent around half of all living vertebrates, comprising over 32,000 extant species (Sallan 2014; Nelson et al. 2016). This remarkable modern diversity has come about through around 400 million years of evolution (Near et al. 2012; Lu et al. 2016; Giles et al. 2017), therefore to fully understand the dynamics of how extant actinopterygians have become so successful, we must turn to the fossil record. The origins of the group can be traced back to the early‐ to mid‐Palaeozoic (Cloutier and Arratia 2004; Lu et al. 2016; Giles et al. 2017), meaning actinopterygians successfully passed through four of the big five mass extinction events of the Phanerozoic (Anderson et al. 2011; Friedman and Sallan 2012; Sallan 2014), events that undoubtedly contributed to shaping the group's evolutionary trajectory. However, studies so far have suggested that none of these events severely detrimentally affected actinopterygians in the same way seen in other clades (Schaeffer 1973; Romano et al. 2014; Friedman 2015; Puttick et al. 2017; Vázquez and Clapham 2017).

The early‐ to mid‐Mesozoic represents a pivotal period in the history of actinopterygians, with the emergence and diversification of neopterygians (Tintori 1998; Tintori et al. 2014a) and subsequent appearance of teleosts (Arratia 2013, 2015; Clarke et al. 2016; Arratia 2017), the group containing over 99% of modern actinopterygian diversity (Nelson et al. 2016). Importantly, the first fossil occurrence of neopterygians occurs just prior to the biggest mass extinction of all time at the end of the Permian, with the earliest known crown group neopterygians appearing in the Early Triassic in the aftermath of this major climatic upheaval (Tintori 1998; Friedman and Sallan 2012; Tintori et al. 2014a; Friedman 2015; Clarke et al. 2016). Teleosts are subsequently thought to have radiated after another, poorly understood extinction event at the end of the Triassic (Sepkoski 1986; Erwin 1995; Arratia 2004; Preto et al. 2010; Friedman and Sallan 2012).

The Permo‐Triassic mass extinction (PTME) is one of the best studied of all extinction events and has been linked to the eruptions of the Siberian Large Igneous Province resulting in massive climatic perturbations potentially analogous to modern anthropogenic climate change (Benton and Twitchett 2003; Kemp et al. 2015). The PTME has been well dated to around 252 Ma (Mundil et al. 2004; Li et al. 2016; Liao et al. 2016). The effects of the PTME on many invertebrate and vertebrate clades have been well documented, at least in terms of species richness (hereafter referred to simply as “diversity”) with many showing dramatic declines across the Permian‐Triassic boundary (PTB) (Jin et al. 2000; Smith and Ward 2001; Retallack et al. 2003; Benton et al. 2004; Benton et al. 2013; Song et al. 2013). The effects on fishes generally, and the Actinopterygii specifically, appear much less dramatic (Schaeffer 1973; Friedman and Sallan 2012; Near et al. 2012; Benton et al. 2013; Romano et al. 2014; Sallan 2014; Puttick et al. 2017; Vázquez and Clapham 2017). Only recently has the effect of the PTME on fishes received strong empirical attention, with studies examining diversity dynamics, body size evolution and rates of extinction across the PTB showing limited and mixed impacts on both osteichthyians as a whole and actinopterygians specifically (e.g., Romano et al. 2014; Puttick et al. 2017; Vázquez and Clapham 2017).

The recovery of marine ecosystems after the PTME marked the beginning of the Mesozoic marine revolution, coincident with the proposed radiation of neopterygians (Vermeij 1977; Kelley and Hansen 2001; Chen and Benton 2012; Friedman and Sallan 2012; Benton et al. 2013). This time period was therefore pivotal in shaping how neopterygians began to diversify, eventually leading to the group's domination of marine ecosystems globally (Friedman and Sallan 2012; Tintori et al. 2014a; Nelson et al. 2016). Around fifty million years after the PTME, another major extinction event, the end‐Triassic mass extinction (ETE), punctuated this neopterygian radiation (Schoene et al. 2010). The ETE has been dated to around 201 Ma, coincident with the Triassic‐Jurassic boundary (TJB) and is again thought to be associated with volcanically induced climate change (Schaltegger et al. 2008; Schoene et al. 2010; Ruhl et al. 2011), but has received far less attention than the PTME (Hallam 2002; Blackburn et al. 2013). The effects of the ETE on life generally are currently poorly understood, especially regarding fish evolution (Schoene et al. 2010; Friedman and Sallan 2012; Blackburn et al. 2013). Teleosts emerged prior to the TJB, in the Middle Triassic (Arratia 2017) but are not thought to have radiated substantially until much later, in the Cretaceous (Friedman and Sallan 2012; Poyato‐Ariza and Martin‐Abad 2016). Understanding the effects of the PTME and ETE on both actinopterygians as a whole, and within each of the major inclusive clades can therefore help uncover the dynamics that led to both the neopterygian and subsequent teleost radiations.

Actinopterygians provide the ideal opportunity to assess the relative impact of both the PTME and ETE on a single clade, as they passed through both events, have one of the best fossil records of any vertebrate clade (particularly in terms of completeness of specimens) and show a wide range of morphologies. Morphometric techniques, the quantification of body shape and important anatomical features, provide a novel way of assessing the relative effects of the PTME and ETE on the group in terms of morphological diversity (disparity). Morphometric techniques and disparity analyses have been applied to several other vertebrate clades throughout the Phanerozoic, and actinopterygians specifically through the Cretaceous‐Paleogene extinction event, 66 My ago, and have provided novel insights not gleaned from diversity studies alone (e.g., Brusatte et al. 2008a,b; Friedman 2009, 2010; Anderson et al. 2011; Thorne et al. 2011; Ruta et al. 2013; Stubbs et al. 2013; Stubbs and Benton 2016).

Here, two aspects of actinopterygian disparity through the Permian to Jurassic interval are investigated. We examine both body shape disparity and jaw functional disparity to determine whether the PTME and ETE resulted in major disparity shifts or morphologically selective extinctions. Any clade negatively affected by a major mass extinction event may be expected to show a marked decrease in any measure of disparity along with diversity, as extinctions across the group reduce the range and diversity of morphologies or ecotypes present. However, in a clade as large as the Actinopterygii, that encompasses multiple ecological guilds, how disparity might change during a mass extinction event or any subsequent recovery period is uncertain. Extinctions may be buffered in terms of overall morphological or functional disparity, despite taxonomic losses within the group as a whole. Conversely, if whole ecological guilds go extinct during these events, a marked decline in disparity would be expected irrespective of whether these losses show a taxonomic signal or not. Within Actinopterygii, three major infraclasses were present in the Permian–Jurassic interval, the Chondrostei, Holostei, and Teleostei (Friedman 2015; Clarke et al. 2016). In addition to overall body shape and lower jaw disparity within the Actinopterygii, we assess trends of these features within the three infraclasses to determine whether any are more or less affected by the extinction events, and to uncover the general pattern of body shape and jaw evolution during the proposed neopterygian and teleostean radiations. Data are also analysed in environmental terms, to determine if any differences in extinction levels are seen in the marine and freshwater realms and to better understand the relative contribution of each environment to the actinopterygian fossil record.

## Materials and Methods

### SAMPLING

Images of actinopterygians were compiled from the literature and photographs taken during visits to the collections of the Natural History Museum, London. A total of 632 individual actinopterygian fossil images were compiled, representing 496 species from 279 genera in 71 actinopterygian families. This sample represents around 39% of described genera from the study interval; taxa omitted are only known from incomplete remains and so could not be assessed using our protocols. A total of 453 whole‐bodied specimens representing 259 genera were used for the geometric body shape analyses, and 365 specimens with well‐preserved skulls representing 222 genera were collated for the jaw functional analyses, although this was later reduced to 219 specimens (see below). A full list of specimens is given in the supplement (Dataset D1).

### TIME BINNING

The sampled taxa range from the earliest Permian (Asselian; 298.9 Ma) to the end‐Jurassic (Tithonian; 145 Ma); a total of 27 stratigraphic stages. The data for both the body shape and jaw functional analyses were initially binned to stage level. However, a paucity of suitable specimens in several stages in the Permian and Jurassic, particularly those of the early‐middle Permian and Middle Jurassic, meant that these bins had relatively low sample sizes. Further time bins were constructed for these low sample bins, by combining short and under sampled stages. Stages immediately before and after the PTB and TJB were initially run as uncombined, to provide precise insights into any morphological or functional changes through the PTME and ETE. A number of taxa could not be assigned specific stage‐level binning in the late Permian and Early Triassic however, and so to ensure the greatest sample size possible, series‐level bins were subsequently run across the PTB.

A total of 17 time bins, comprising 12 stages and five combined time bins, were used for the disparity analyses: early Permian; middle Permian; late Permian; Early Triassic; Anisian; Ladinian; Carnian; Norian; Rhaetian; Hettangian; Sinemurian; Pliensbachian; Toarcian; Middle Jurassic; Oxfordian; Kimmeridgian; Tithonian. The minimum bin sample size was five for the geometric body shape analyses and two for the jaw functional analyses, both in the middle Permian, a series renown for having few fish faunas preserved globally (Friedman and Sallan 2012; Friedman 2015). Jaw functional disparity was not calculated for the middle Permian. In the disparity time‐series we plotted bin sample size as a (within‐study) diversity measure and to allow direct comparisons between sample size and disparity.

### BODY SHAPE DISPARITY—GEOMETRIC MORPHOMETRIC PROTOCOLS

**Figure 1:**
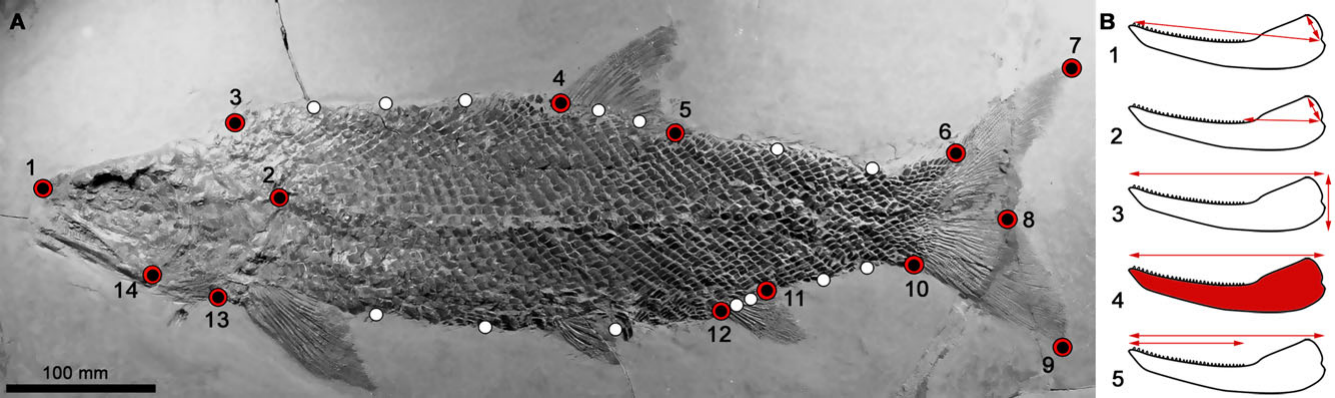
(A) The landmark scheme used to capture the body shape of actinopterygians for the geometric morphometric analyses. Fourteen fixed landmarks were placed at discrete anatomical features (numbered red and black points). Fourteen semilandmarks capture the curvature of the body between important fixed landmarks (white points). Points are numbered according to the order in which they are landmarked, with semilandmarks placed after fixed landmarks in a clockwise direction from point (3) until point (13). (B) The measurements taken from lower jaws for the functional analyses. Ratios were taken from the following measurements to generate functional variables: 1. Anterior mechanical advantage (MA)–inlever (jaw joint to adductor attachment point) divided by the outlever (jaw joint to anterior‐most teeth). 2. Posterior MA—inlever (as above) divided by the outlever (posterior‐most teeth). 3. Maximum jaw depth–max jaw depth divided by jaw length. 4. Average jaw depth –jaw area (without teeth) divided by jaw length twice. 5. Relative tooth row length–tooth row length divided by jaw length. [Color figure can be viewed at wileyonlinelibrary.com]

We quantified actinopterygian body shape disparity using geometric morphometrics. The database of actinopterygian whole‐body images was used to digitize two classes of two‐dimensional landmarks using the software tpsDig 2.0 (Rohlf 2015): (1) primary landmarks at fixed points on discrete, morphological features, and (2) secondary (semi) landmarks that capture curvature between important primary landmarks to represent overall body shape (Mitteroecker and Gunz 2009; Gunz and Mitteroecker 2013). We asses shape variation in the lateral body profile with landmarks (Fig. 1A) identified based on Friedman (2010) and by observation of all specimens, to determine the most informative morphological features. A total of 14 fixed primary landmarks and 14 semilandmarks were used, marking six curves around the body between important fixed landmarks (Supplementary methods; Fig. 1A). Distal fin rays were not used as landmark points and were excluded from the analysis, as few specimens preserve the complete extent of either paired or unpaired fins, and the wide variance in fin position postmortem would not allow accurate comparisons. Caudal fins appear less prone to these issues and thus could be accurately captured by fixed landmarks. While most specimens were preserved completely and in life‐orientation, a number of taxa were only represented by distorted specimens. Two procedures were employed to mitigate these distortions based on the type of deformation as set out by Friedman (2010) and are described in detail in the supplementary information.

Body shape morphospace was constructed to explore major aspects of shape variability. The landmark coordinate data were subjected to generalized Procrustes analysis using tpsRelw (Rohlf 2014), removing the noise effects of size and orientation. Bending energy minimization criteria was used to slide the semilandmarks. A principal component analysis (PCA) was then performed on the procrustes aligned data using the plotTangentSpace function in the Geomorph R package (Adams and Otárola‐Castillo 2013). This assimilated shape variation into a set of principal components, each capturing major components of variation. A total morphospace of all taxa was produced from the first two principal components. Morphospaces were also constructed for each series, to visualize changes in disparity through time. We illustrate shape changes associated with the first two principal components using thin‐plate spline deformation plots, these were created using the plotRefToTarget function from the R package Geomorph (Adams and Otárola‐Castillo 2013).

### JAW DISPARITY—FUNCTIONAL MORPHOMETRIC PROTOCOLS

Jaw functional disparity was measured using five continuous characters (Fig. 1B), each known to relate to feeding and utilized in previous studies (Westneat 2003; Anderson et al. 2011; Smithwick 2015). Only measurements from the lower jaws were taken, as this allowed the greatest number of individuals to be included due to the propensity for this feature to be preserved in fossil specimens. The feeding system of actinopterygians has been shown to be well represented by lower jaw mechanics that can be calculated from the overall jaw shape, justifying its use as a functional and ecomorphological proxy (Westneat 2003; Anderson et al. 2011). The functional character measurements are: (1) anterior mechanical advantage (MA); (2) posterior MA; (3) maximum jaw depth/length; (4) average jaw depth/length; (5) relative dental row length (Fig. 1B). These functional measurements have been comprehensively described elsewhere (e.g., Westneat 2003; Anderson et al. 2011; Smithwick 2015). Further functional measures were considered, but preservational restrictions precluded many, particularly those of the dentition and posterior region of the mandible that is often covered by a maxilla in fossils and a full dentition rarely exposed. Being unable to assess characters based on the dentition is a limitation, as jaws showing similar shapes may have different dentitions adapted to different feeding styles. Measurements were taken from photographs using the software ImageJ (Abràmoff et al. 2004). Species represented by multiple well‐preserved specimens had their functional characters averaged from all measured individuals. Measurements from all taxa are provided in the Dataset D1.

A multidimensional functional morphospace was constructed from the jaw character data. Originally, the five measurements were recorded from all 365 jaw specimens. However, poor preservation of the tooth row and obscuration of the posterior region of the mandible by the maxilla resulted in missing data (17.2%), primarily for characters two and five. We first considered imputing missing values with a regularized iterative PCA algorithm using the function imputePCA from the R package FactoMineR (Husson et al. 2016). However, the resulting morphospaces had taxa positioned linearly along principal component two, with near identical scores on that axis of variation (Fig. S1). This could distort further disparity calculations, and it did not represent a biological signal. We therefore opted to perform our primary analyses on the 219 jaw specimens with all characters recorded. For this, we used a standard PCA to identify major elements of functional variation and produce morphospaces using the principal components scores. Jaw functional morphospaces were constructed for all taxa and for temporal series‐level bin divisions.

### TEMPORAL DISPARITY TRENDS

Disparity can be viewed as both the density of morphospace occupation, incorporating the dissimilarity and spread of taxa, and as the overall volume or expanse of morphospace occupation (Ciampaglio et al. 2001; Kotrc and Knoll 2015; Pigot et al. 2016). Saturation or packing in morphospace may reduce the average dissimilarity between forms, but the extent of overall morphospace occupied may remain stable. Here, we explore both aspects of disparity. We also performed sensitivity analyses with sampled taxa known only from Lagerstätten deposits removed. All disparity calculations were performed in R, using custom scripts modified from Hughes et al. (2013) and Kotrc and Knoll (2015), and in MATLAB (The MathWorks), using the package MDA (Navarro 2003).

To quantify morphospace density, we calculated the within‐time‐bin sum of the variances (henceforth variance) across principal component axes. All axes were used for both body shape temporal disparity (56 axes) and jaw functional disparity (five axes). For a sensitivity test in the body shape analyses, we also calculated within‐time‐bin variance from variable numbers of principal component axes, beginning first with two axes (59% variance), then five axes (81% variance), 20 axes (99% variance), and finally from all 56 axes (100% variance). Disparity trends are consistent in all the calculations (Fig. S2). We also calculated the mean pairwise dissimilarity (MPD) from Procrustes distances between specimens in each time bin, to explore variation from the aligned landmark data. For both the variance and MPD calculations, we used 1000 bootstrap replicates to generate 95% confidence intervals around the mean disparity value for each time bin.

The volume of morphospace occupied through time was measured using the convex hull volume metric. For both body shape disparity and jaw functional disparity, we calculated the within‐time‐bin convex hull volume for each bin using increasing numbers of principal component axes, firstly based on axes one and two (area), eventually incorporating dimensions one through to five (hypervolume). So volumes calculated from a variable number of principal component axes could be compared, we standardized each temporal disparity series by dividing volume in each time bin by the largest value in each time series.

### PERMUTATION TESTS

We used permutation tests to examine the statistical significance of changes in morphospace occupation and disparity metrics across the PTB and TJB. To test for significant shifts in morphospace occupation, we used nonparametric multivariate analysis of variance (NPMANOVA) (Anderson 2001). NPMANOVA tests the equality of multivariate group means (centroid positions) based on permutation tests. All principal components were used to represent both body shape and jaw morphospace occupation. The Euclidean distances between time bin means were first computed and then compared to distances generated through random permutation of time bin assignments (with 9999 replications). Using a similar approach, we also test for significant differences in the calculated variance, MPD and PCO convex hull volume in time bins across the PTB and TJB. To test the null hypothesis of no differences in calculated disparity, we compare observed differences to randomized differences in disparity using permutation tests with 1000 time bin membership randomizations. For both tests, statistically significant differences are denoted by *P*‐values <0.05.

### PARTIAL DISPARITY—TAXONOMIC AND ENVIRONMENTAL TRENDS

The methods described above considered actinopterygian disparity as a single inclusive global sample. We also investigated trends linked to disparity in subclades and from different environments, to provide both sampling and macroevolutionary insights. For this, we divided our samples into three infraclasses: Holostei, Chondrostei, and Teleostei (inclusive of known stem taxa; i.e., Teleosteomorpha, sensu Arratia 2001, hereafter referred to as teleosts) and an *Incertae sedis* grouping used for taxa with uncertain taxonomic affinity, and freshwater and marine environmental groupings (based on the lithologies of the fossils). We used the partial disparity metric from Foote (1993) to calculate the amount of overall disparity in each time bin that is attributable to a given taxonomic and environmental subgroup, for both body shape and jaw function.

## Results

### MORPHOLOGICAL VARIATION AND MORPHOSPACE OCCUPATION

**Figure 2:**
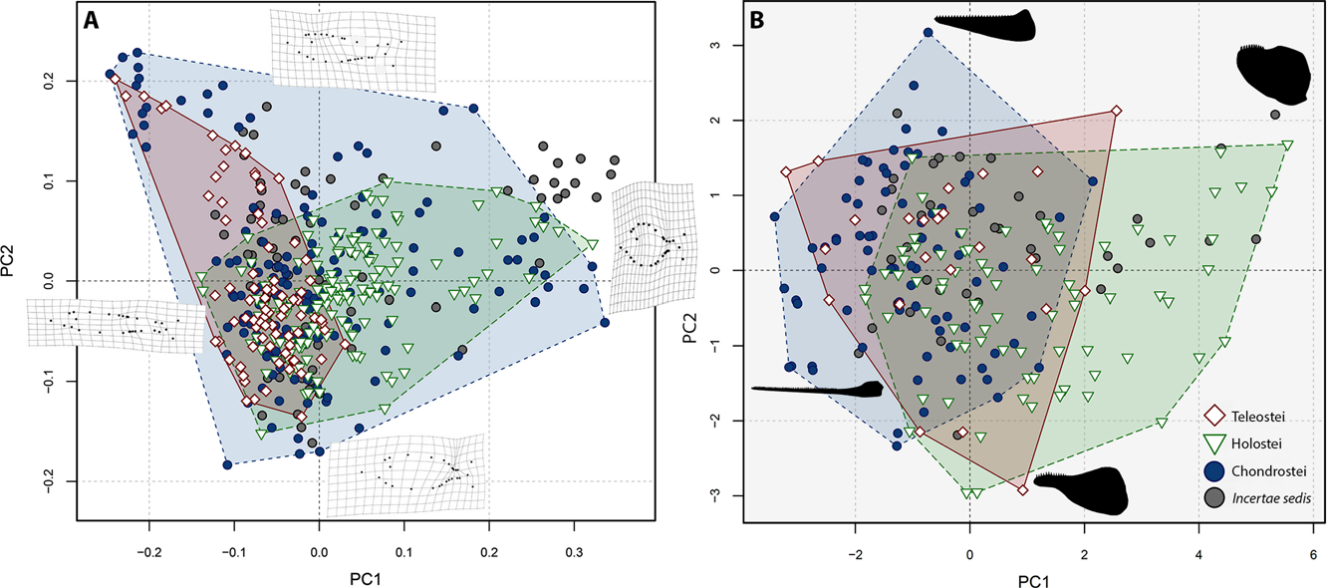
Total geometric morphospace (A) and functional morphospace (B) for all sampled actinopterygians from the Permian‐Jurassic divided by infraclass. In A, PC1 represents 39% of the variation and PC2 represents 20%. In B PC1 represents 68% of the variation and PC2 represents 24%. Thin plate spline grids illustrate the extreme shapes (maximum and minimum) of principal component (PC) axes 1 and 2 for the body shape morphospace (A). Illustrations of the most disparate observed lower jaw morphologies are depicted on the functional morphospace highlighting the taxa in which they are found (B). These jaws represent the following taxa; PC1 minimum–*Saurichthys*, PC1 maximum–*Dapedium*, PC2 minimum–*Sangiorgioichthys*, PC2 maximum–*Coccolepis*. [Color figure can be viewed at wileyonlinelibrary.com]

The PCA on body shape landmarks produced 56 principal component axes. The first five principal components account for 81% of the variance, and the first two axes subsume 59% of variance, and will be used to qualitatively describe morphospace occupation (Dataset D1). PC1 (39%) resolved body depth and elongation as being the most significant biological signal, while PC2 (20%) represents the position of the dorsal fin as being the second most important morphological feature (Fig. 2A). These results conform to those found in disparity analyses of fishes through the Cretaceous‐Paleogene (Friedman 2010), and of modern reef fish disparity (Claverie and Wainwright 2014), indicating the importance of these features in actinopterygian morphology throughout their history.

All jaw functional variation is represented by five principal component axes, and the first two axes account for 92% of variance (Dataset D1). Principal component loadings show that PC1 (68%) accounts for variation in the anterior MA, maximum jaw depth relative to jaw length and average jaw depth relative to jaw length (Fig. S3). These characters are known to be related to feeding modes in fishes, such as durophagy and piscivory (Westneat 2003; Friedman 2010; Anderson et al. 2011; Smithwick 2015). PC2 (24%) subsumes variation in the relative length of the dental row and posterior MA (Fig. S3).

Body shape and jaw functional morphospaces reveal several noteworthy trends. In body shape morphospace, chondrosteans occupy almost all areas, showing a wide range of morphologies positioned at the extremities of both PC1 and PC2 (Fig. 2). It should be noted however, that we included the order Saurichthyiformes in Chondrostei based on Wu et al. (2013), but this placement is not universally agreed upon and could change with revised phylogenetic analyses in the future (Tintori et al. 2014b). This would have important implications for the body shape analyses as the order is represented by the slenderest body shapes seen throughout the study period. Holosteans and teleosts each occupy a smaller region within the chondrostean total space, but are partially separated from one another (Fig. 2). Holosteans expand into morphospace representing deep bodied forms (high positive PC1 scores). In contrast, teleosts are largely confined to negative PC1 scores, showing more elongate morphologies. A cluster of *Incertae sedis* represents the still enigmatic pycnodonts (Poyato‐Ariza 2015) showing high positive scores on both PC 1 and 2, highlighting their deep‐bodied morphology.

Jaw functional morphospace shows some key differences to body shape trends. Chondrosteans do not have such expansive occupation, and do not overlap all other taxa (Fig. 2). Each infraclass occupies a similar total area, but only chondrosteans and holosteans explore any unique regions of functional morphospace. Approximately half of holostean functional disparity lies within a distinctive functional morphotype, with high positive PC1 scores, representing deep and robust jaws with high anterior MA. The only other taxa within this space are the pycnodonts. Surprisingly, teleosts do not expand into any unique areas of functional morphospace, rather exploring areas already represented by the other infraclasses (Fig. 2).

### TEMPORAL DISPARITY TRENDS

**Figure 3:**
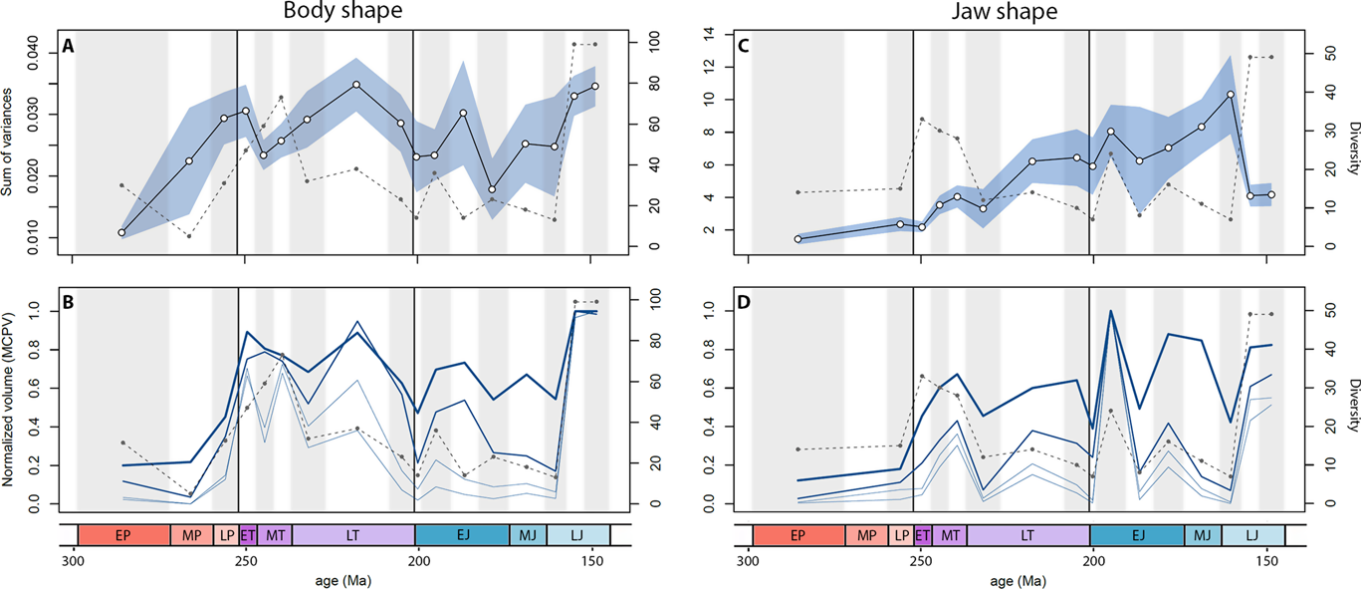
Within‐bin disparity time series for geometric (body shape; A and B) and functional (jaw shape; C and D) variance and normalized morphospace volume (minimum convex polygon volume—MCPV). Variance (A and C) is calculated from all geometric (56) and functional (five) PC scores. The mean variance for each bin is plotted as the midpoint per time bin, with 95% confidence intervals represented by the light blue area. The weight of blue lines for the MCPV plots (B and D) indicate the number of PC axes represented, from two (heaviest line) to five (lightest line). Diversity (sample size; dashed line) is plotted alongside each disparity measure. Vertical black lines indicate the Permo‐Triassic mass extinction event (PTME) at 252 Ma and the end‐Triassic extinction (ETE) at 201 Ma. Abbreviated series names: E = Early; M = Middle; L = Late; P = Permian; T = Triassic; J = Jurassic. [Color figure can be viewed at wileyonlinelibrary.com]

**Figure 4:**
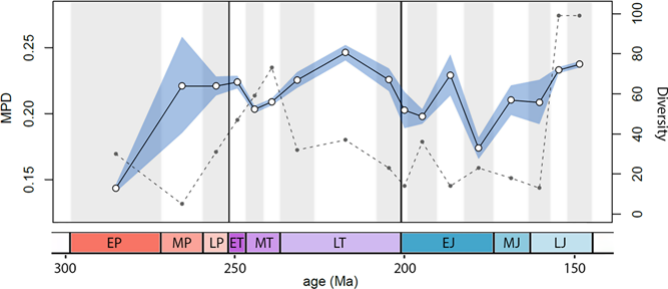
Geometric body shape disparity time series, measured as within‐bin mean pairwise Procrustes distances (MPD). Mean MPD is plotted at the midpoints for each bin and 95% confidence intervals are represented by the light blue area. Diversity (sample size) is represented by the dashed line. [Color figure can be viewed at wileyonlinelibrary.com]

Density‐based metrics for actinopterygian body shape disparity show no significant changes through the PTME and ETE. Disparity was greatest in the late Permian, Early Triassic, and stages of the Late Triassic and Late Jurassic. Trends based on the variance and MPD are very similar (Figs. 3A and 4). Disparity is lowest in the early Permian and rises toward the PTB. No change is observed across the PTB, and this is robustly supported by permutation tests (variance *P* = 0.781, MPD *P* = 0.825). A slight reduction in disparity is seen in the Middle Triassic. From a disparity peak in the Norian, it then falls in both the Rhaetian, prior to the TJB, and in the Hettangian. The disparity decline across the TJB is not statistically significant (variance *P* = 0.374, MPD *P* = 0.303). With the exception of the Pliensbachian, disparity remains relatively low in the Early Jurassic, before steadily rising to a Late Jurassic high (Figs. 3A and 4). There is no clear relationship between bin disparity and diversity (sample size).

**Figure 5:**
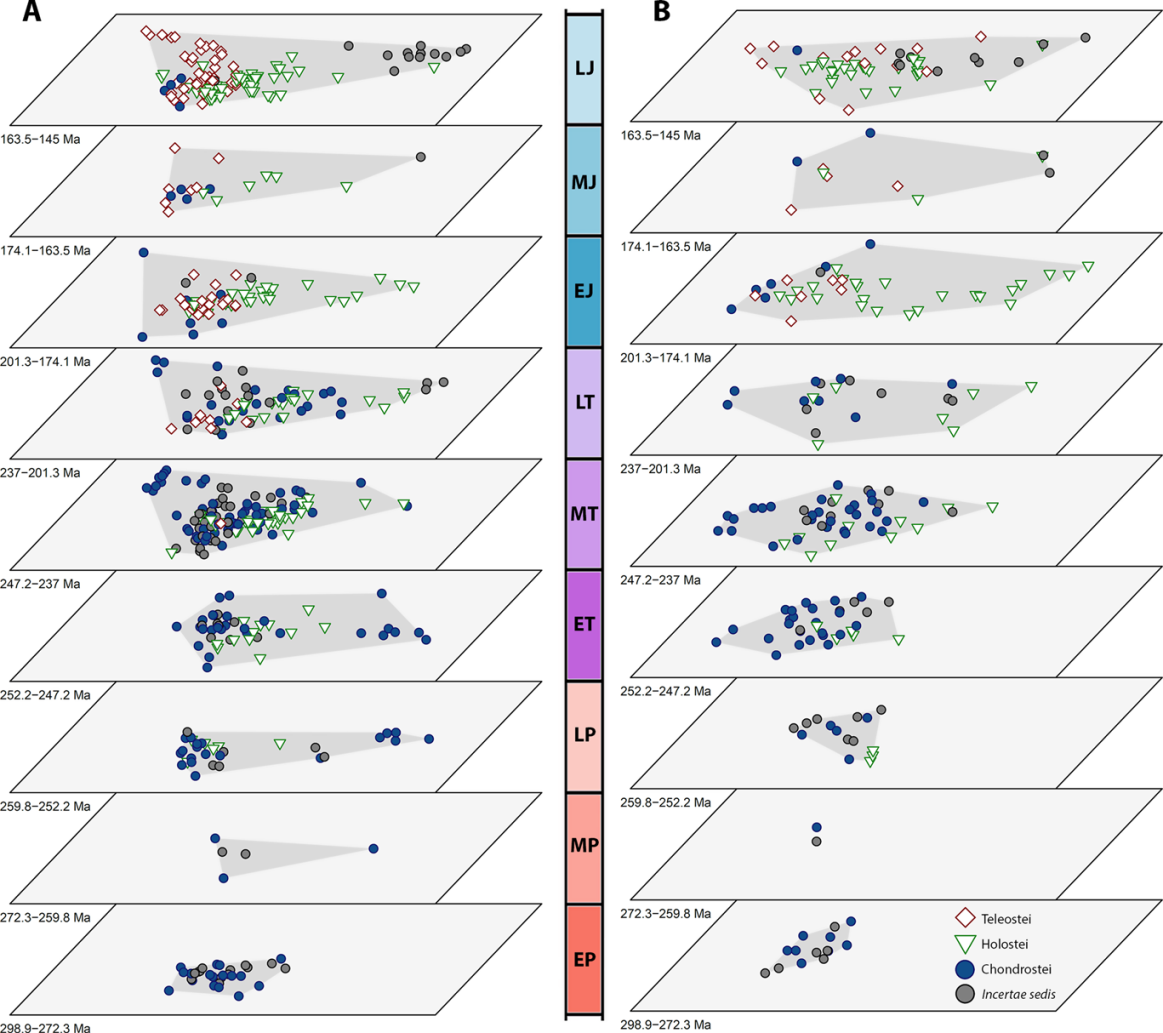
The pattern of actinopterygian geometric morphospace (A) and functional morphospace (B) occupation through time divided by series, from the early Permian (bottom) to the Late Jurassic (top), based on the first two PC axes. Data are separated by infraclass. Both time series show expansions from low space occupation in the Permian to high in the Jurassic, however geometric morphospace occupation expands more sharply and earlier in the Triassic than that of functional morphospace. Abbreviated series names: E = Early; M = Middle; L = Late; P = Permian; T = Triassic; J = Jurassic. [Color figure can be viewed at wileyonlinelibrary.com]

Trends of body shape morphospace volume through time reveal that morphospace occupation was most expansive in the Late Jurassic (Figs. 3B and 5A). However, in agreement with the density‐based metrics, high disparity was achieved relatively early, with an expansion of morphospace volume seen in the Early Triassic followed by a steady expansion up to the Late Jurassic peak (Figs. 3B and 5A). Notably, in contrast to the density‐based metrics, there is a marked increase in volume between the late Permian and Early Triassic. Permutation tests show this increase in disparity is statistically significant, whether morphospace volume is quantified using two axes (area) (*P* = 0.047) or five axes (*P* = 0.026). In contrast, the volume reduction across the TJB is not statistically significant (two axes *P* = 0.974, five axes *P* = 0.781). Through both extinction intervals there are no significant shifts in body shape morphospace occupation (NPMANOVA: *P*‐values ranging from 0.076–0.899, see Dataset D1). When assessed at infraclass level, Chondrosteans account for most of the morphospace occupied throughout the Permian and Triassic (Fig. 5A). Holosteans expand in the Middle and Late Triassic, after which time they remain relatively static in morphospace. Chondrosteans morphospace diminished during the Late Triassic and Jurassic, resulting in holosteans occupying a unique region of morphospace in the Early and Middle Jurassic (Fig. 5A). Teleosts expand through the Late Triassic and Jurassic, occupying a unique area of morphospace by the Late Jurassic. Pycnodonts (*Incertae sedis*; (Poyato‐Ariza 2015)) take over the deep‐bodied region of morphospace in the Late Jurassic, previously occupied by both chondrosteans and holosteans. This highlights a pattern observed regularly where similar regions of morphospace are occupied through time, but different taxa account for this morphology. Interestingly however, this region is never explored by teleosts within our study period, which instead generally cluster in areas indicative of more slender body shapes (Fig. 5A).

Density‐based metrics for jaw functional disparity through time show different patterns to body shape disparity. An overall long‐term trend of increasing disparity through time is recovered, with exceptionally low disparity in the Permian and a peak in the Early to Middle Jurassic (Fig. 3C). No major shifts are observed across either extinction boundary (PTB variance *P* = 0.572, TJB variance *P* = 0.847). A major drop in disparity is seen from the Oxfordian to Kimmeridgian and Tithonian bins, coinciding with a big spike in diversity (Fig. 3C). This decline in the variance is unlikely to represent a genuine loss of overall functional variation in actinopterygians. Instead, it represents a saturation of functional morphospace during these stages when diversity substantially increased and many taxa possessed similar jaw morphologies. This conclusion is supported by examining jaw functional morphospace volume, where the overall volume of functional morphospace occupied during the Kimmeridgian and Tithonian remains high, almost comparable the maximal level for the time series (Fig. 3D). Functional morphospace volume through time also shows a general trend of increasing jaw disparity, from low levels in the Permian, intermediate disparity in the Triassic, to consistently high disparity the Jurassic (Figs. 3D and 5). A notable expansion occurs from the Early to Middle Triassic (Fig. 5B). There are no significant changes in jaw functional morphospace volume across either extinction boundary (*P*‐values ranging from 0.092–0.971, see Dataset D1). Similarly, through both extinction intervals, there are no significant changes in functional morphospace occupation (NPMANOVA: *P*‐values range from 0.104–0.66, see Dataset D1). The Early Jurassic witnessed the radiation of holosteans into an area not widely explored in any previous series. This region corresponds to characters known to relate to durophagy (high mechanical advantage and increased jaw depth; Smithwick 2015). From the Middle Jurassic onwards, this space becomes occupied by the pycnodonts.

For both body shape and jaw functional disparity, the removal of taxa known only from Lagerstätten had little effect for all metrics (Fig. S4). Only negligible differences were found in the Middle Triassic. Importantly, little change was observed in the Late Jurassic, when many taxa come from the Lagerstätten deposits around Solnhofen, Germany (Lambers 1999; Arratia et al. 2015).

### INFRACLASS PARTIAL DISPARITY

**Figure 6:**
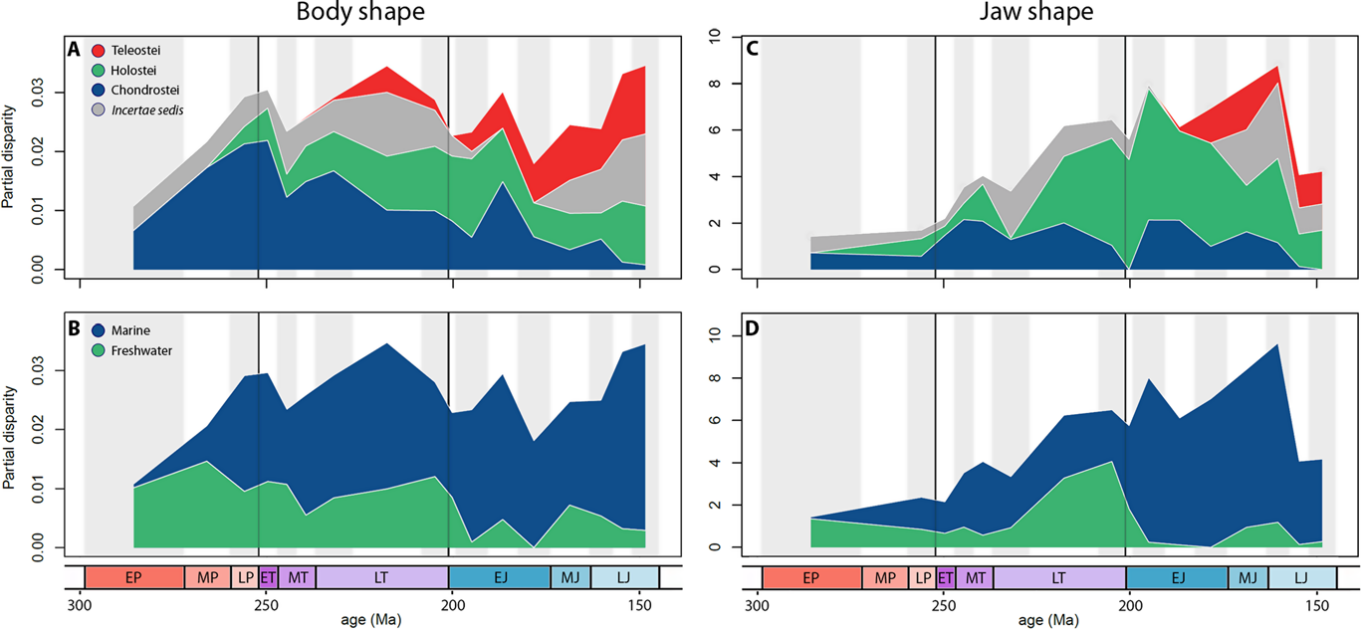
Partial disparity showing the relative contribution of clades (infraclasses) and environment (marine vs freshwater) to total disparity. (A) The relative contribution of each infraclass to total geometric (body shape) disparity. (B) The relative contribution of each environment to total geometric disparity. (C) The relative contribution of each infraclass to total functional (jaw shape) disparity. (D) The relative contribution of each environment to total functional disparity. Abbreviated series names: E = Early; M = Middle; L = Late; P = Permian; T = Triassic; J = Jurassic. [Color figure can be viewed at wileyonlinelibrary.com]

Partial disparity illustrates the relative contribution of each clade (infraclass) to total body shape and jaw functional disparity through time (Fig. 6A and C). In body shape disparity, chondrosteans make the greatest contribution to disparity until the Late Triassic, when holosteans and teleosts begin to expand (Fig. 6A). After the TJB, chondrosteans continue to decline in their relative contribution to disparity and the other major clades expand further, particularly the teleosts from the Early to Late Jurassic. In the Late Jurassic, the large contribution from *Incertae sedis* comes from the pycnodonts. No major perturbations or turnovers are observed across either extinction boundary.

Partial functional disparity provides insights to both clade dynamics and sampling. The major contributor to overall functional disparity is the holosteans, that dominate the Late Triassic and Jurassic (Fig. 6C). Chondrosteans contribute a smaller proportion of functional disparity when compared to the body shape analysis. The drop‐off of holosteans and sudden increase in *Incertae sedis* taxa in the Ladinian hints that many of the poorly described specimens may well be holosteans, based on their abundance and disparity contribution before and after this time bin, and the relative static contribution of chondrosteans. Interestingly, teleosts disparity contributions are relatively small, and they only reach a maximum of around one‐third of partial functional disparity by the end of the Jurassic (Fig. 6C). Again, a high contribution of *Incertae sedis* in the Late Jurassic represents the pycnodonts. Across the PTB and TJB, no clades show a drop in partial disparity. The apparent decline of chondrosteans to zero into the Hettangian is a sampling artefact, as the clade reemerges to its highest levels of functional disparity in the Sinemurian.

### MARINE AND FRESHWATER DISPARITY

Freshwater taxa are the dominant contributors to disparity in both the early and middle Permian (Fig. 6B and D), highlighting the fact that most deposits containing fish fossils from this time are freshwater (Friedman and Sallan 2012). In the Triassic, marine taxa dominate body shape disparity trends (Fig. 6B). Partial functional disparity in the Triassic shows that freshwater taxa contribute relatively little until the Norian and Rhaetian, where their contribution substantially increases to over 50% (Fig. 6D). In the Jurassic, marine taxa dominate both measures of disparity, with freshwater taxa barely contributing to functional variation throughout. Patterns of partial disparity are not driven by sample size in either marine or freshwater taxa (Fig. S5).

## Discussion

### ACTINOPTERYGIANS ACROSS THE PTME

The PTME is thought to have resulted in the extinction of up to 96% of all marine species and around 49% of terrestrial tetrapod families (Raup 1979; Benton and King 1989; Benton and Twitchett 2003; Benton et al. 2013). While these levels of severity are generally seen for groups such as marine invertebrates (e.g., Wang and Sugiyama 2000; Payne 2005; Twitchett and Oji 2005; Brayard et al. 2009), fishes appear to have passed across the PTB relatively unscathed in most previous analyses (Schaeffer 1973; Thomson 1976, 1977; Benton 1998; Orchard 2007; Reguant 2007; Friedman and Sallan 2012; Romano et al. 2014; Friedman 2015; Vázquez and Clapham 2017). Fossil fish deposits are however notoriously poor in the Permian, which appears to have made most assessments of the PTME and its impacts on fishes difficult (Friedman and Sallan 2012; Friedman 2015). The lack of suitable fossil deposits in the Permian is thought to be related to the palaeogeography at the time. The paucity of marine facies in the early‐middle Permian, as observed in our data, was likely due, at least in part, to the destruction of coastlines during the formation of the supercontinent Pangaea (Friedman and Sallan 2012). A marked increase in marine facies is seen from the late Permian onwards however (Fig. 6). Throughout the Permian, the data here show a general expansion of morphologies despite diversity dropping, possibly linked to the poor sampling at this time, and a static pattern of functional evolution (Figs. 3, 4, 5, 6). It seems therefore, that either ecospace was not being expanded despite new body types emerging, or that the very low sample size in the middle Permian (two for the functional analyses) could be depressing the true levels of functional disparity. A restricted ecotype range has been suggested previously at this time, attributed to a lack of ecological opportunities and available habitat in the Permian (again likely related to the formation of Pangaea) and therefore the results here may represent a genuine pattern irrespective of sample size (Friedman and Sallan 2012). The pattern may only be better discerned with future fossil discoveries from the Permian.

A recent study examining body size trends and diversity dynamics of fishes through the PTME showed decreases in body size of freshwater taxa and elevated turnover rates at the PTB, but extinction rates were broadly in line with background levels before and after the PTME (Romano et al. 2014; Friedman 2015). Romano et al. (2014) further suggested that a taxonomic turnover occurred across the PTB within marine apex predatory guilds, indicating higher trophic levels suffered from the PTME. While we did not attempt to specify trophic guilds in our data, the lack of shifts in any measure of functional disparity suggest that no major changes in feeding modes occurred, at least at our time bin resolution.

Another recent study examining extinction rates and body size dynamics across the PTB found elevated phylogenetic signal of extinctions across the boundary, but body length played no role in differential survival or extinction rates (Puttick et al. 2017). As size was not analyzed in our study we cannot comment on any changes across the PTB, but our findings of a slight increase in morphospace volume and no significant changes in density‐based disparity measures suggest minimal morphological change across the boundary. In terms of taxonomic losses within our study data, a single order appears to go extinct at the PTME, the Dorypteriformes (Cope 1871), with the constituent Dorypteridae representing one of two family‐level losses along with the Elonichthyidae. While this only takes into account the 39% of genera represented by well‐preserved specimens in this study, more severe losses would be expected even in a reduced dataset if the PTME had the severe negative effects observed in other major clades. Therefore, we find no evidence of marked losses either morphologically or taxonomically.

Our results contrast a predicted pattern of major biodiversity losses across the PTB, with slight increases in measures of body shape variance along with diversity gains and a significant expansion of body shape morphospace (Figs. 3, 4, 5, 6). It therefore appears that the diversity and disparity of actinopterygians when analyzed at our time resolution was not negatively affected by the PTME. This may corroborate the idea that a group as large as actinopterygians containing many ecological guilds may be buffered against severe losses during mass extinction events. Alternatively, the bias of the fossil record between a poorer record of complete fossil specimens in the Permian and a richer record in the Triassic (Tintori et al. 2014a) could be causing an apparent diversity and disparity increase as an artefact of sampling, as suggested by others (e.g., Vázquez and Clapham 2017). Further, our temporal resolution may not be fine enough to detect any negative impacts. If severe reduction in body shape or functional disparity and a subsequent recovery occurred within the four million years comprising the Early Triassic, it could be hidden from our current analyses, suggesting a rapid recovery from any losses incurred at the PTB. The lack of major taxonomic losses at the PTB however suggest that actinopterygians were not affected as negatively as many other major groups.

### TRIASSIC ACTINOPTERYGIANS AND THE NEOPTERYGIAN RADIATION

Although the PTME appears not to have negatively affected actinopterygians, some interesting patterns occur throughout the Triassic, at a time when ecosystems globally were thought to be recovering from the devastating effects of the PTME (Benton and Twitchett 2003; Benton et al. 2013). Body shape variance appears to reduce from the Early Triassic into the Middle Triassic (Fig. 3A and B), at a time when previous assumptions on marine ecosystem recovery would suggest they should be expanding. Morphospace occupation at this time however does confirm that the range of morphologies increases into the Middle Triassic, but with many taxa clustering in saturated areas (Fig. 5), which likely explains the apparent drop off in the variance and MPD. Overall, a general trend of expansion in body shape disparity is seen throughout the Triassic until a drop‐off in the Rhaetian. This trend is mirrored in the jaw functional data, where a steady increase in functional disparity is seen in all measures, particularly in the Late Triassic, however no drop is seen in the Rhaetian. These results correspond to the proposed radiation of neopterygians (Benton et al. 2013; Romano et al. 2014; Friedman 2015). Although molecular estimates suggest that neopterygians arose as early as the Carboniferous, or even late Devonian (Santini et al. 2009; Near et al. 2012; Betancur‐R et al. 2013; Broughton et al. 2013; Friedman 2015), the first unequivocal crown group neopterygians appear soon after the PTME, and previous work has implied a radiation in the Middle to Late Triassic (Romano et al. 2014; Friedman 2015). This important diversification appears to have occurred as conditions became more favorable after the PTME and it may have been the case that vacant ecospace, left by other marine taxa that did not survive the PTME, became occupied by new neopterygian taxa (Benton and Twitchett 2003; Benton et al. 2013).

The novel functional morphospace expansion toward deep jawed, high MA taxa (indicative of durophagy) in the Middle and Late Triassic (Fig. 5) suggests the evolution of new feeding strategies and movement into new niches, which occurs around the time proposed for the radiation of the neopterygians (Tintori 1998; Romano et al. 2014; Friedman 2015). Previous work has suggested that evolutionary novelties in the jaw apparatus of neopterygians, allowing feeding modes such as durophagy, began to emerge in the Late Triassic, which is supported by our data (Tintori 1998; Lombardo and Tintori 2005; Romano et al. 2014). Durophagous actinopterygians showing novel jaw mechanics are suggested to have emerged for the first time in the Middle to Late Triassic, becoming common by the Norian (Tintori 1998; Lombardo and Tintori 2005). In our data, areas of functional morphospace indicative of high MA and deep jaws are indeed expanded into at this time (Fig. 5). In the Late Triassic, novel feeding modes evolved that were not only related to overall jaw morphology, but also in neopterygian dentition (e.g., Gibson 2015). Specific features of the dentition were not considered in our data, however the expansion of jaw morphologies seen in our results and novel dentition types found by others highlight that neopterygians were evolving completely new ecotypes in the Middle‐Late Triassic.

The morphological expansion of actinopterygians through the Middle to Late Triassic also coincides with expansion in jaw functional disparity in marine reptiles, which were the likely main predators of many fishes at the time (Stubbs and Benton 2016). Increased predation pressures could have helped drive an expansion into new niches, such as benthic durophagous habits, away from the water column where most marine reptiles would have hunted. While this is currently speculative, a potential coevolution between neopterygians and marine reptiles may have been underway throughout the latter stages of the Triassic and warrants further examination. An interesting feature to note however is the importance of freshwater facies for actinopterygians in the Middle‐Late Triassic (Fig. 6), suggesting that it was not only in the marine realm that neopterygians were evolving novel jaw morphologies. A further group that undoubtedly had influence on the pattern of morphospace and ecospace occupation of actinopterygians is the chondrichthyans, a clade that also passed through the PTME and ETE with apparent minimal losses of diversity or disparity (Friedman and Sallan 2012; Koot 2013; Romano et al. 2014).

The oldest members of total group Teleosteomorpha are found in the late Middle Triassic of China and Italy (Tintori et al. 2015; Arratia 2017). While the diagnosis and interrelationships of both stem and crown group teleosts are still contentious and under revision (Arratia 2004, 2017), the earliest members of the clade appear to have shown conservative morphologies nested well within the known morphospace of chondrosteans (Fig. 5). Unfortunately, due to the skull anatomy of these early teleosts, few taxa could have all five functional characters measured and thus had to be omitted, resulting in an insufficient sample size in the functional analyses to assess the earliest stages of the group's functional evolution, a feature that warrants further study.

### TRANSITION THROUGH THE ETE

Estimates of the effects of the ETE on life have so far been difficult to assess, with most data coming from marine invertebrates (Hallam 2002). Faunal turnovers and evolutionary bottlenecks have however been observed in some vertebrate taxa such as ichthyosaurs (Thorne et al. 2011). As with the PTB, there is a paucity of exposures spanning the TJB, and many of the fossils from the stage immediately preceding the boundary (the Rhaetian) are fragmentary (Storrs 1994; Ward et al. 2001; Foffa et al. 2014).

The picture of actinopterygian evolution through the TJB shown here is not clear‐cut, but as with the PTB it seems that actinopterygians were not detrimentally affected by the ETE. Small drops across the boundary in jaw functional and body shape variance are no greater than between a number of other successive time bins, and no reductions are observed in functional morphospace or body shape morphospace. Despite no major reductions across the TJB, the Sinemurian appears to be a time of high diversity and functional disparity, with no concomitant increase in body shape disparity. Much of the increase in functional disparity in the Sinemurian is the expansion of taxa with deep jaws and high MA, indicated by high scores on PC1 (Figs. 3 and 5). These taxa were likely durophages based on their jaw morphologies (Smithwick 2015). While deep‐jawed, presumed durophages appear to have been important components in actinopterygian faunas since the neopterygian radiation in the Middle‐Late Triassic, the jaw morphologies seen in the Sinemurian expand the functional morphospace into areas not seen at any other time. It is possible that new niches were being explored in the wake of the ETE, based upon expansion of preexisting morphologies that had evolved in the Late Triassic (Tintori 1998). This is exemplified by the expansion of the family Dapediidae in the Early Jurassic, particularly with the genus *Dapedium* that is thought to have been a generalist durophage with a body plan similar to that seen in other taxa in the Late Triassic but with exceptionally deep jaws providing very high MA ratios (Tintori 1998; Smithwick 2015). The family's unique jaw morphology (Fig. 1B), which may have been even better adapted to durophagy than those of other Middle and Late Triassic taxa, seems to have opened up new opportunities in the wake of the ETE, allowing expansion of the clade in the Early Jurassic (Tintori 1998; Smithwick 2015).

Across the TJB, two orders appear to go extinct from within our dataset, the Perleidiformes and Scanilepiformes. Only families constituent to these orders are lost, and so extinctions at higher taxonomic levels seem minimal, as with the PTME, and much lower than would be expected for a severe extinction event.

### ACTINOPTERYGIAN EVOLUTION THROUGH THE JURASSIC

The Pliensbachian‐Toarcian is thought to have been a time of climatic disturbances, with the Toarcian Oceanic Anoxic Event and a possible second‐order extinction event occurring (Little and Benton 1995; Caruthers et al. 2013). This could explain the fluctuations seen here in the Pliensbachian and Toarcian, but the event could not be tested in more depth because creating time bins shorter than stages was not possible with the current data and these events were likely much shorter in duration than the more major extinction events (Little and Benton 1995). The Jurassic marks the point at which teleosts begin to constitute significant components of the overall actinopterygian evolutionary picture. Teleosts maintain a relatively limited range of body shapes in the Early Jurassic, but expand into novel areas of morphospace in the Middle and Late Jurassic that were previously occupied by chondrosteans in the Triassic (Fig. 5). One of these areas is that representative of long, slender body shapes occupied by the basal teleosteomorph aspidorhynchiforms (Arratia 2001, 2004, 2013, 2017), that were previously exhibited by the Saurichthyiformes. While the placement of Saurichthyiformes within Chondrostei (Wu et al. 2013) is not universally accepted, they are generally thought to be non‐neopterygian (Kogan and Romano 2016), and thus the Middle‐Late Jurassic is the first occurrence of neopterygians showing this distinct elongate body shape. Functionally, teleosts also expand markedly into the Late Jurassic although low sample sizes in the Middle Jurassic make the nuances of this expansion hard to discern. By the Late Jurassic, teleosts were beginning to explore novel functional morphospace outside of that observed in the other infraclasses (Fig. 5), potentially linked to the novel jaw mechanics that teleosts evolved (Motta 1984; Clarke et al. 2016), although this exploration appears minor at this time, and teleosts only account for a third of the observed partial disparity (Fig. 6C). This is at a key time in teleostean evolution, when important extant lineages such as the elopomorphs and ostariophysans first appear in the fossil record (Arratia 1997, 1999, 2010). It has been suggested that although teleosts began to become more prevalent in actinopterygian faunas in the Late Jurassic, they did not radiate expansively until the Cretaceous (Clarke et al. 2016; Poyato‐Ariza and Martin‐Abad 2016). Our data appears to support this, and suggests that the clade was only beginning to explore novel functional morphospace minimally in the Late Jurassic and expanded into morphospace previously occupied by chondrosteans (Figs. 5 and 6).

The Late Jurassic represented a time of high morphological disparity in actinopterygians (Figs. 3, 4, 5, 6). During this interval teleosts and holosteans were diverging to explore different areas of morphospace (Fig. 5), a pattern that may have set the stage for teleosts to supersede holosteans later in the Cretaceous and become the dominant actinopterygian clade until the present day. One group of note that may alter this pattern however is the enigmatic Pycnodontiformes (Poyato‐Ariza 2015). This group of deep‐bodied, mostly presumed durophagous fishes has long been contentious in its phylogenetic placement within Actinopterygii (Poyato‐Ariza 2015). Despite often being assigned as stem group teleosteans, pycnodonts have recently been suggested as basal neopterygians not representing stem teleosts or holosteans (Poyato‐Ariza 2015). While their phylogenetic affinities are beyond the scope of this article, their placement within either major neopterygian infraclass would markedly affect the pattern of both body shape and functional disparity, particularly in the Late Jurassic, as pycnodonts cluster within both body shape morphospace and functional morphospace mostly occupied by holosteans, far from any known teleosts (Fig. 5).

The peaks in sample size in the Late Jurassic correspond with the increase in total fish diversity observed in previous work, with the high abundance of exceptional fossil sites yielding fishes at this time (e.g., Solnhofen, Germany and Cerin, France (Friedman and Sallan 2012)) cited as a potential explanation. Our removal of Lagerstätten only taxa from the data set made little difference to the overall body shape or jaw functional disparity Fig. S4.

## Conclusions

Suggestions that the PTME and ETE did not have the severe impacts on actinopterygians seen in other clades are borne out in this study. Little change is observed in either body shape or jaw function across either extinction boundary, other than a slight increase in body shape morphospace volume. This holds true not only for overall actinopterygian disparity, but also for each major infraclass and taxa from the marine and freshwater realms. During the Middle‐Late Triassic, expansion of body shape and jaw function is likely linked to the radiation of neopterygians, with novel feeding modes and body plans associated with the evolution of new ecotypes, particularly durophages. Across the TJB, as many of the Late Triassic durophagous neopterygians disappeared, the family Dapediidae radiated into new ecospace likely due to their unique combination of novel jaw mechanics in preexisting body plans. Fluctuations in diversity and disparity through the Early Jurassic, possibly linked to environmental disturbances, precede a steady expansion into the Late Jurassic, as teleosts began to become more prevalent. The teleostean radiation likely occurred later however, in the Cretaceous, as only minimal divergence between teleosts and holosteans is observed in the Jurassic. This study confirms, with numerical evidence, that ray‐finned fishes were remarkably resistant, as a clade, to the massive environmental perturbations from two of the greatest mass extinctions of all time.

### AUTHOR CONTRIBUTIONS

F.S devised the project and gathered and compiled the data. T.S performed the statistical analyses of the data. Both authors wrote the manuscript.

Associate Editor: M. Zelditch

Handling Editor: M. Noor

## Supporting information


**Figure S1**. Impact of missing data on the functional morphospace.
**Figure S2**. Mean within‐time bin variance calculated from variable numbers of PC axes.
**Figure S3**. Principal component loadings for the functional PCA analysis. PC1 (68%) accounts for variation in the anterior mechanical advantage (MA), maximum jaw depth and average jaw depth. PC2 (24%) accounts for variation in relative dental row and posterior MA.
**Figure S4**. Effect of Lagerstätten removal from disparity time series.
**Figure S5**. Environmental partial disparity vs sample size.
