# Supplementary material for: Phanerozoic survivors: Actinopterygian evolution through the Permo‐Triassic and Triassic‐Jurassic mass extinction events
Source: Evolution. 2018 Feb 2;72(2):348–62. doi: 10.1111/evo.13421 (PMC5817399; doi:10.1111/evo.13421)
Supplement: Supplementary file 1 — Figure S1. Impact of missing data on the functional morphospace. Figure S2. Mean within‐time bin variance calculated from variable numbers of PC axes. Figure S3. Principal component loadings for the functional PCA analysis. PC1 (68%) accounts for variation in the anterior mechanical advantage (MA), maximum jaw depth and average jaw depth. PC2 (24%) accounts for variation in relative dental row and posterior MA. Figure S4. Effect of Lagerstätten removal from disparity time series. Figure S5. Environmental partial disparity vs sample size. [file EVO-72-348-s001.docx]

Supplementary Information for **Phanerozoic survivors: actinopterygian evolution through the Permo-Triassic and Triassic-Jurassic mass extinction events**

***SUPPLEMENTARY METHODS***

**GEOMETRIC LANDMARKING**

A total of 14 fixed primary landmarks was used (Fig. 1A) on all images which were oriented in one direction. The primary landmark scheme was as follows for all specimens: **1**. the tip of the premaxilla; **2**. the posterior-most point of the braincase meeting the vertebral column (estimated from the position of the operculum in specimens with a covering of thick scales); **3**. the supraoccipital crest; **4**. dorsal fin anterior-most fin ray insertion; **5**. dorsal fin posterior-most fin ray insertion; **6**. principal fin ray insertion of the caudal fin; **7**. caudal fin principal ray distal point; **8**. posterior-most point of the central fin ray in the caudal fin; **9**. caudal fin ventral-most fin ray distal point; **10**. ventral-most fin ray insertion of caudal fin; **11**. anal fin posterior-most fin ray insertion; **12**. anal fin anterior-most fin ray insertion; **13**. pectoral girdle ventral-most tip; **14**. lower jaw joint. A total of six curves were measured using semi-landmarks (Fig. 1A).

**CORRECTING DISTORTED SPECIMENS**

Individuals showing post-mortem contortion, common in fishes and resulting from muscle contraction-induced curvature of the body along the long axis (Friedman 2010), were retrodeformed using the software tpsUtil (Rohlf 2013). Specimens showing imperfect preservation of morphological features, such as caudal fins were digitally reconstructed using Photoshop Elements 5 (Adobe Systems, San Jose, California, USA) depending on the severity and location of fragmentation. Caudal fin incompleteness was the most common form of fragmentation, and was mitigated by aligning multiple partial specimens of the same species, mirroring of the preserved lobe in taxa thought to have homocercal tails, or by estimation of landmark positions from well-preserved closest relative taxa. These approaches are thought to be the most conservative approach to including taxa only known from incomplete material, and thus may miss exceptional morphologies not present in fossils (Friedman 2010).

***SUPPLEMENTARY FIGURES***

**
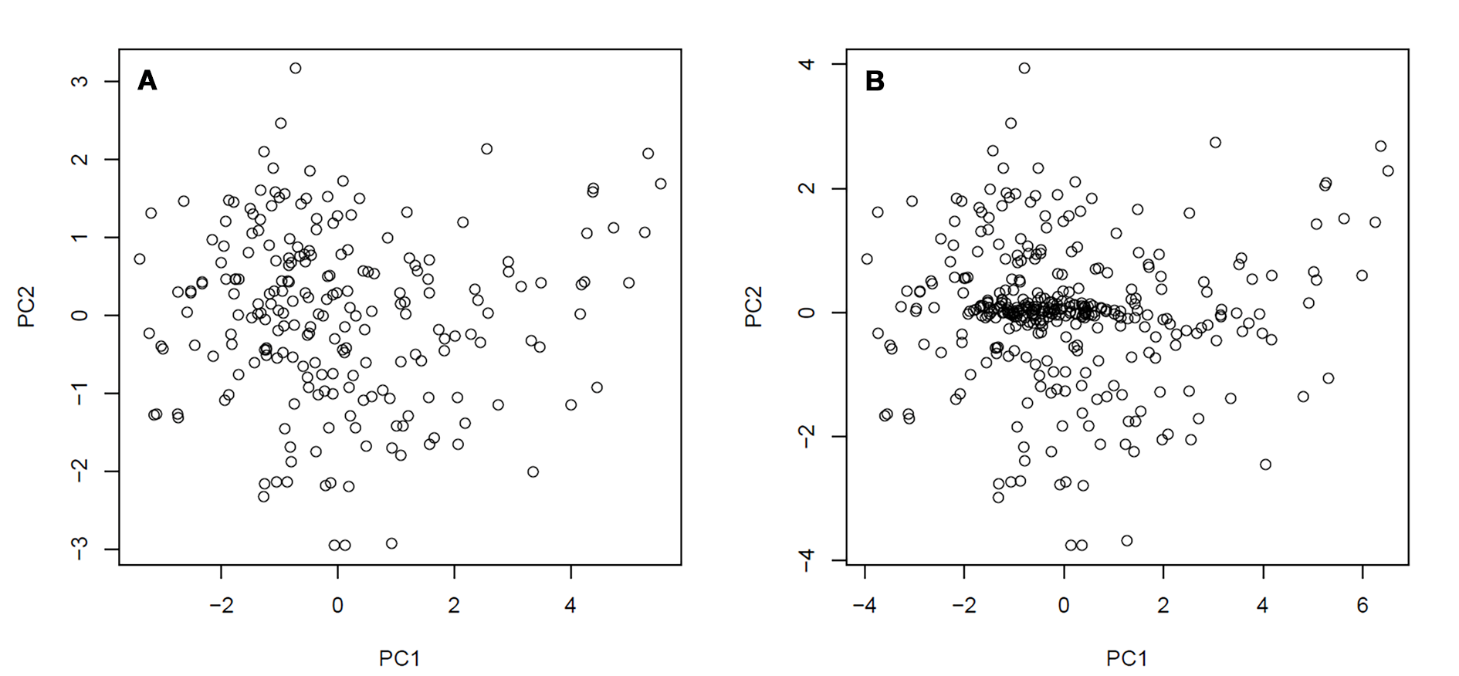
Figure S1.** Impact of missing data on the functional morphospace. Two morphospace plots are shown with taxa showing missing data removed (**A**) and with missing characters estimated using a regularised iterative PCA algorithm (**B**). When missing characters were estimated, specimens were plotted linearly along PC2 (B). As this does not represent a true biological signal and would impact upon further disparity analyses this method was dismissed and instead specimens with missing data were omitted from the final analyses.


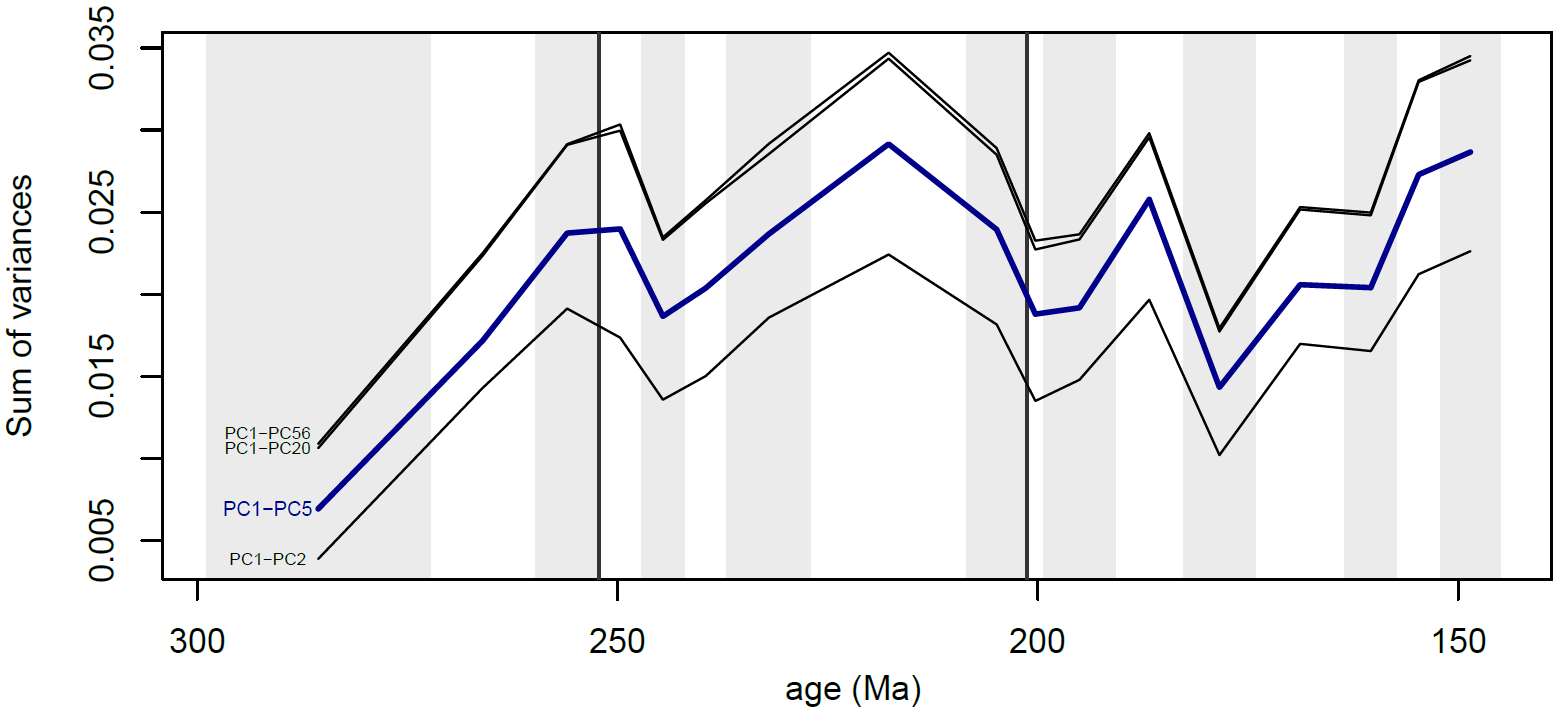


**Figure S2.** Mean within-time bin variance calculated from variable numbers of PC axes. Variance curves are shown for the first two axes (59% variance), five axes (81% variance), 20 axes (99% variance), and all 56 axes (100% variance). The overall relative pattern is consistent across all analyses.


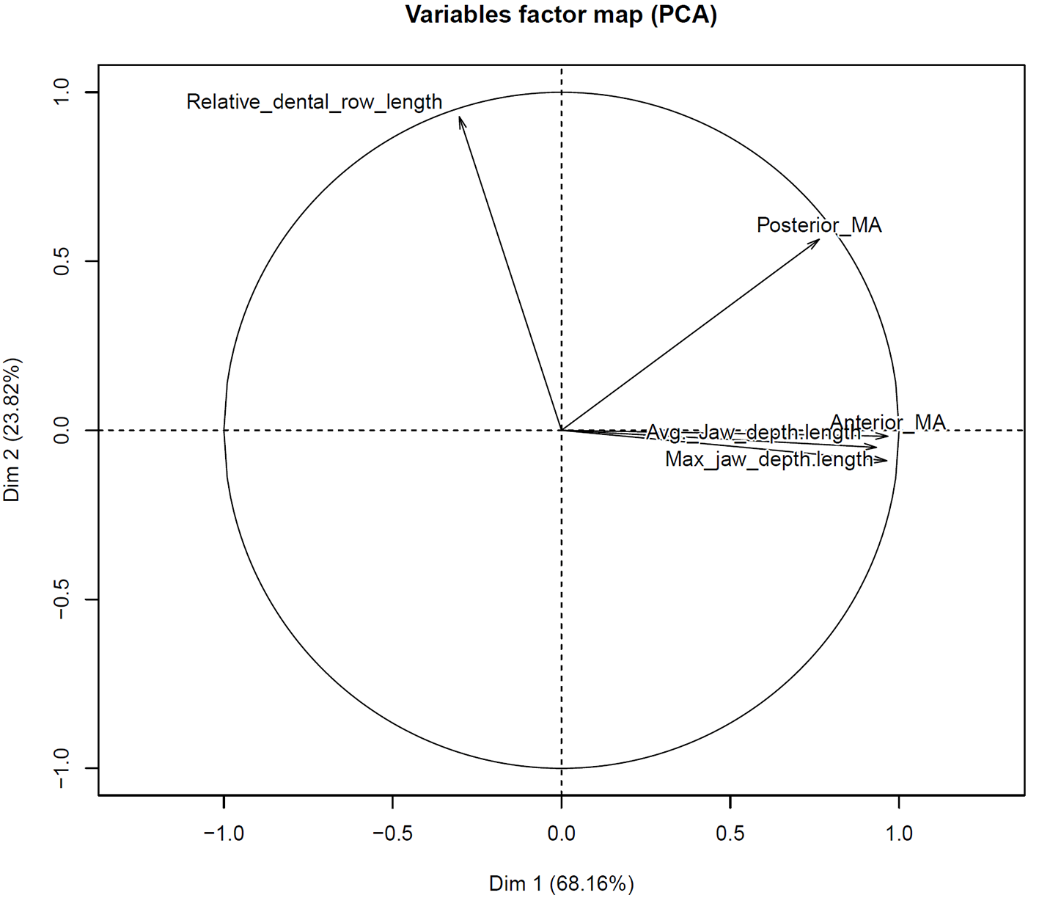


**Figure S3.** Principal component loadings for the functional PCA analysis. PC1 (68%) accounts for variation in the anterior mechanical advantage (MA), maximum jaw depth and average jaw depth. PC2 (24%) accounts for variation in relative dental row and posterior MA.


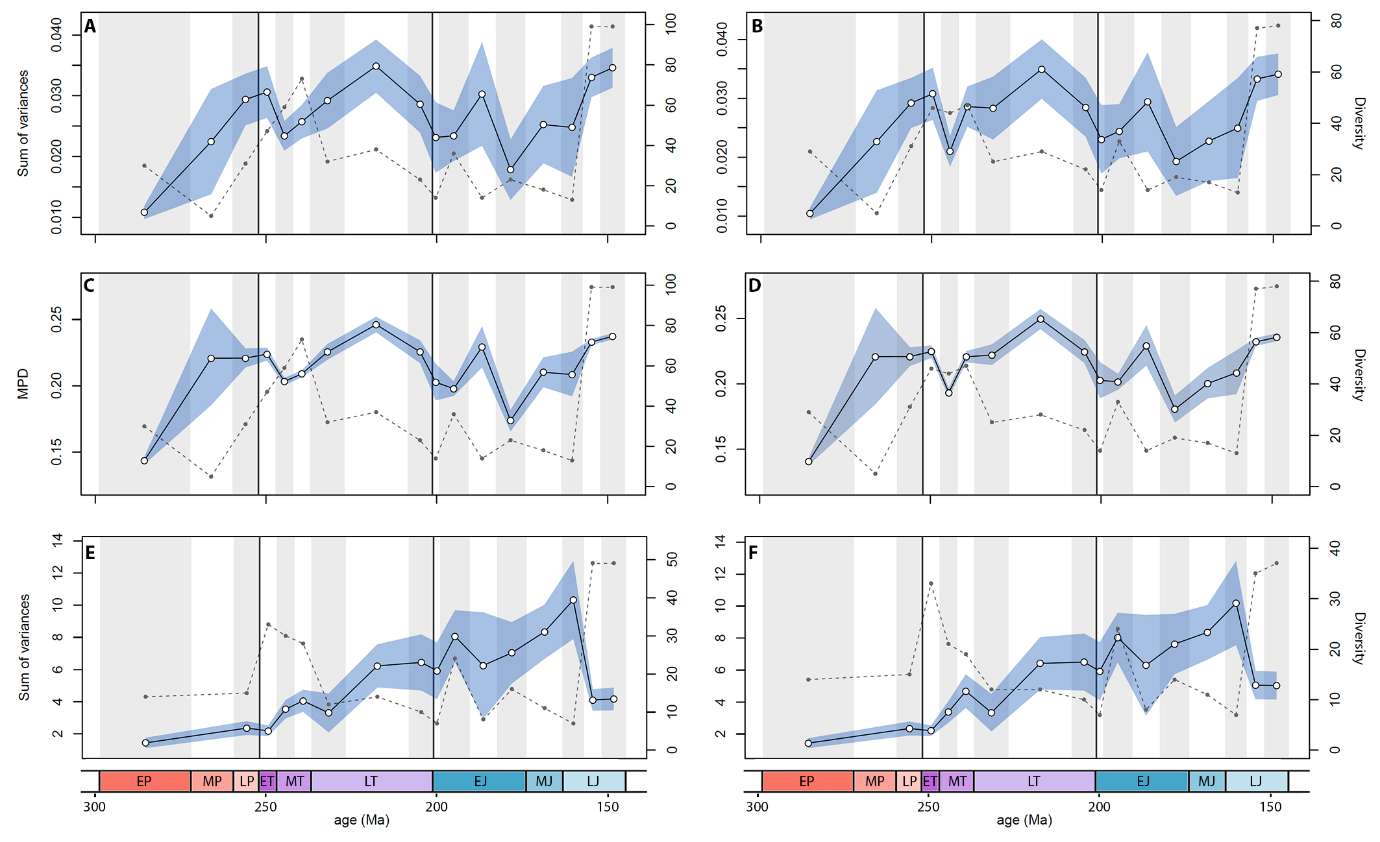


**Figure S4.** Effect of Lagerstätten removal from disparity time series. **A**. Geometric (body shape) variance with Lagerstätten included. **B**. Geometric variance with Lagerstätten excluded. **C**. Geometric mean pairwise distances (MPD) with Lagerstätten included. **D**. Geometric MPD with Lagerstätten excluded. **E**. Functional variance with Lagerstätten included **F**. Functional variance with Lagerstätten excluded. Little difference occurs when Lagerstätten are removed from any time series, indicating that the uneven distribution of exceptional fossil sites through time does not alter the general disparity patterns observed in actinopterygians.


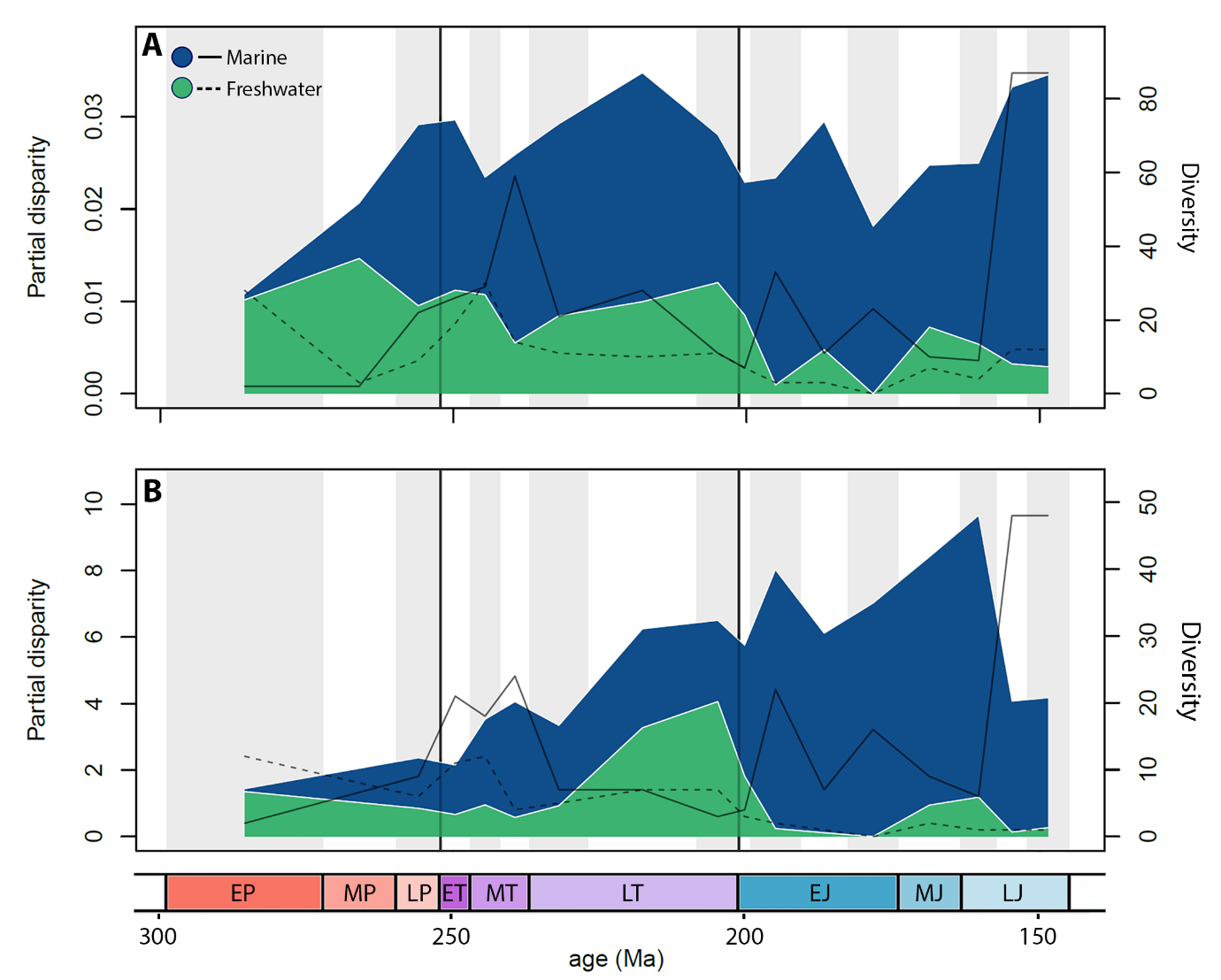


**Figure S5.** Environmental partial disparity vs sample size. Generic diversity (sample size) is plotted (marine – solid line; freshwater – dashed line) alongside partial disparity for marine and freshwater environments. Apart from a small number of time bins where sample size and partial disparity for each environment appear correlated, there is an overall general disconnect between the two, indicating that the relative contribution of marine and freshwater disparity is not primarily driven by sample size alone.

***SUPPLEMENTARY REFERENCES***

Friedman, M. 2010. Explosive morphological diversification of spiny-finned teleost fishes in the aftermath of the end-Cretaceous extinction. Proceedings of the Royal Society of London B: Biological Sciences:rspb20092177.

Rohlf, F. J. 2013. tpsUtil., Department of Ecology and Evolution, State University of New York at Stony Brook, United States of America.
